# Supplementary material for: Defining the host dependencies and the transcriptional landscape of RSV infection
Source: mBio. 2025 Aug 15;16(9):e01010-25. doi: 10.1128/mbio.01010-25 (PMC12421885; doi:10.1128/mbio.01010-25)
Supplement: Supplemental Figures — Figures S1 to S6. [file mbio.01010-25-s0002.docx]

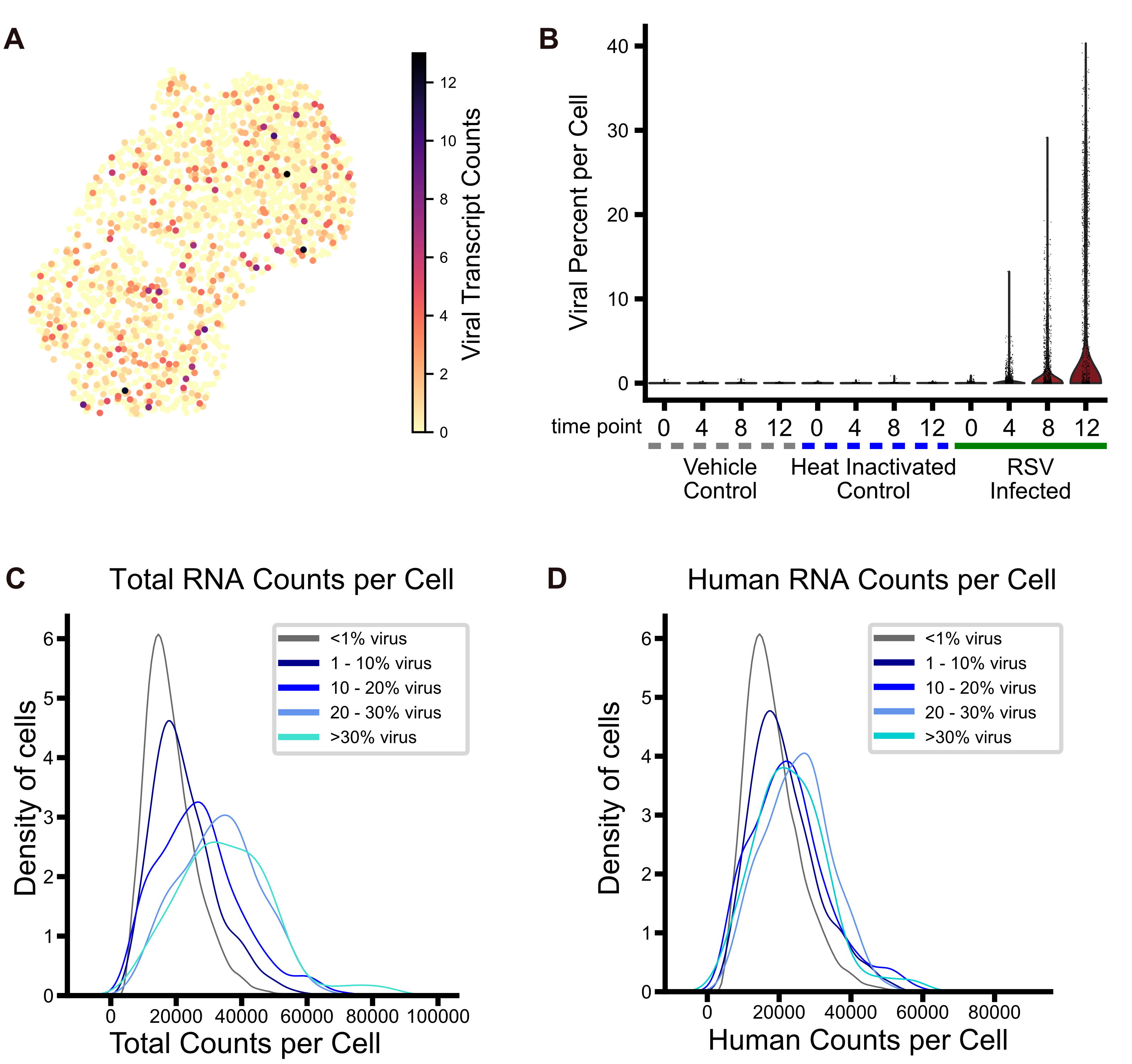


**Figure S1: Evaluation of viral gene expression and distribution of RNA counts per cell.**

**A.** To evaluate ambient viral RNA captured in droplets during single-cell RNA sequencing, we included a spike-in of murine cells and quantified viral transcript counts per cell. **B.** The viral percentage per cell for each condition and time point was calculated. **C-D.** Cells were binned by percentage of viral transcripts and each plot represents the density for (**C**) total counts and (**D**) human counts per cell.


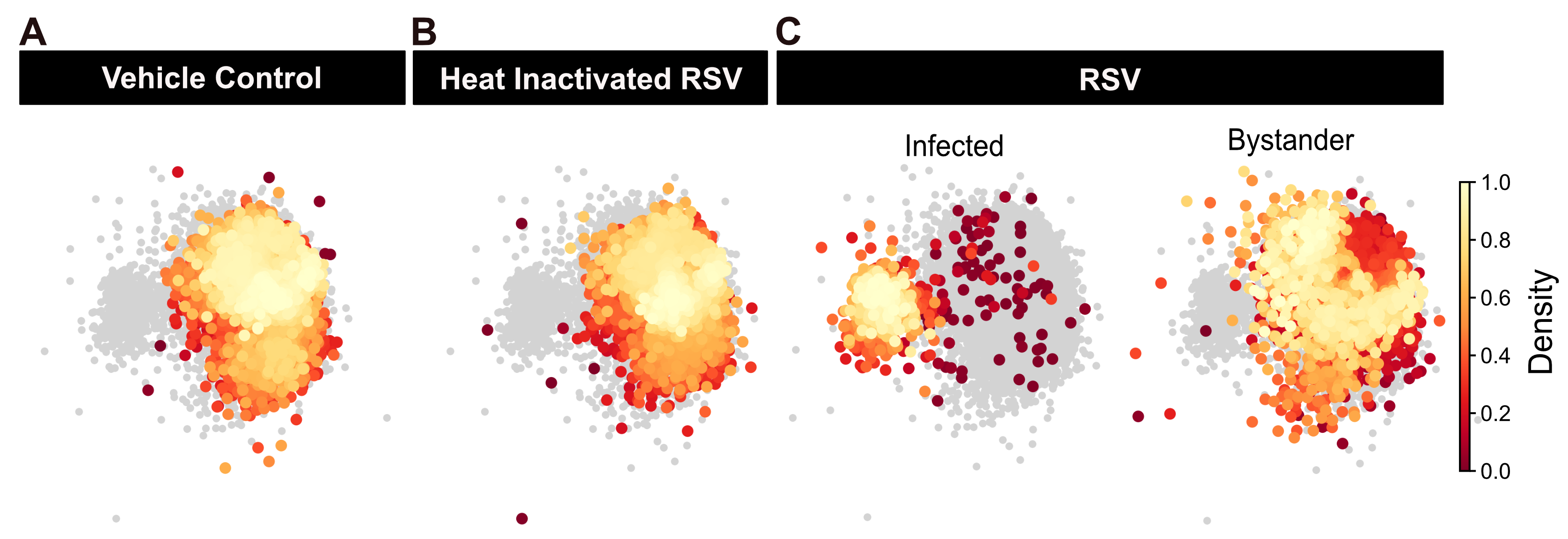


**Figure S2: Single-cell transcriptomes projected into UMAP space with density-preserving visualization method.**

**A-C**. Projection of single-cell transcriptomes into UMAP space with incorporation of density-preserving visualization method (densMAP (20)) for the 12 hour time point. The density of cells for each treatment condition are overlaid onto all cells for this time-point (gray). Cells treated with RSV (**C**) were broken down by infected or bystander cells.


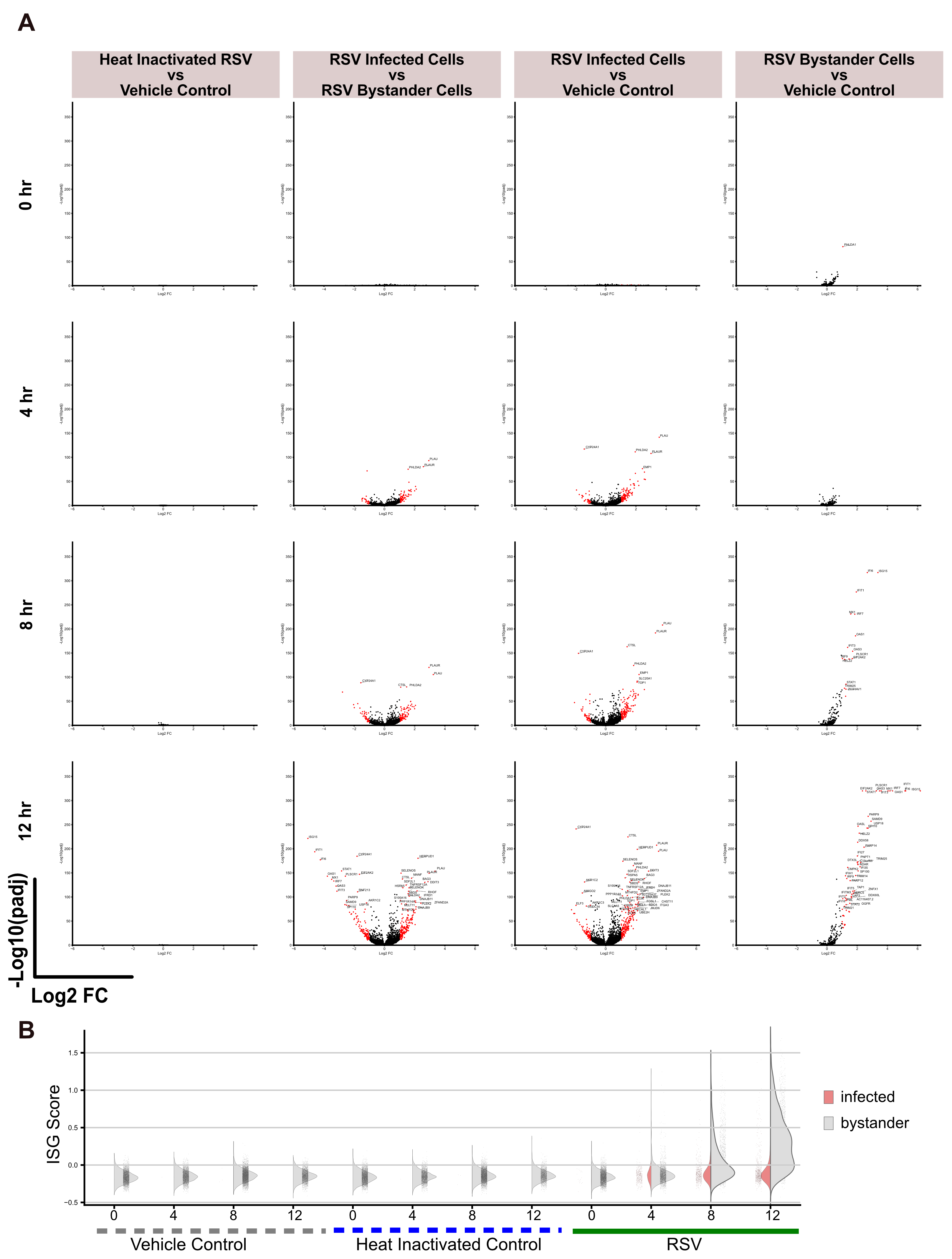


**Figure S3: Differential expression results for each time point and condition.**

**A.** For each time point and treatment condition, we performed differential expression analysis (MAST). Resulting volcano plots show fold change (FC) and adjusted *P*-values for each pairwise comparison. **B.** Each cell was scored by expression of interferon-stimulated genes (ISGs), and ISG score for each time point and condition is shown for infected cells (pink) and bystander cells (gray).


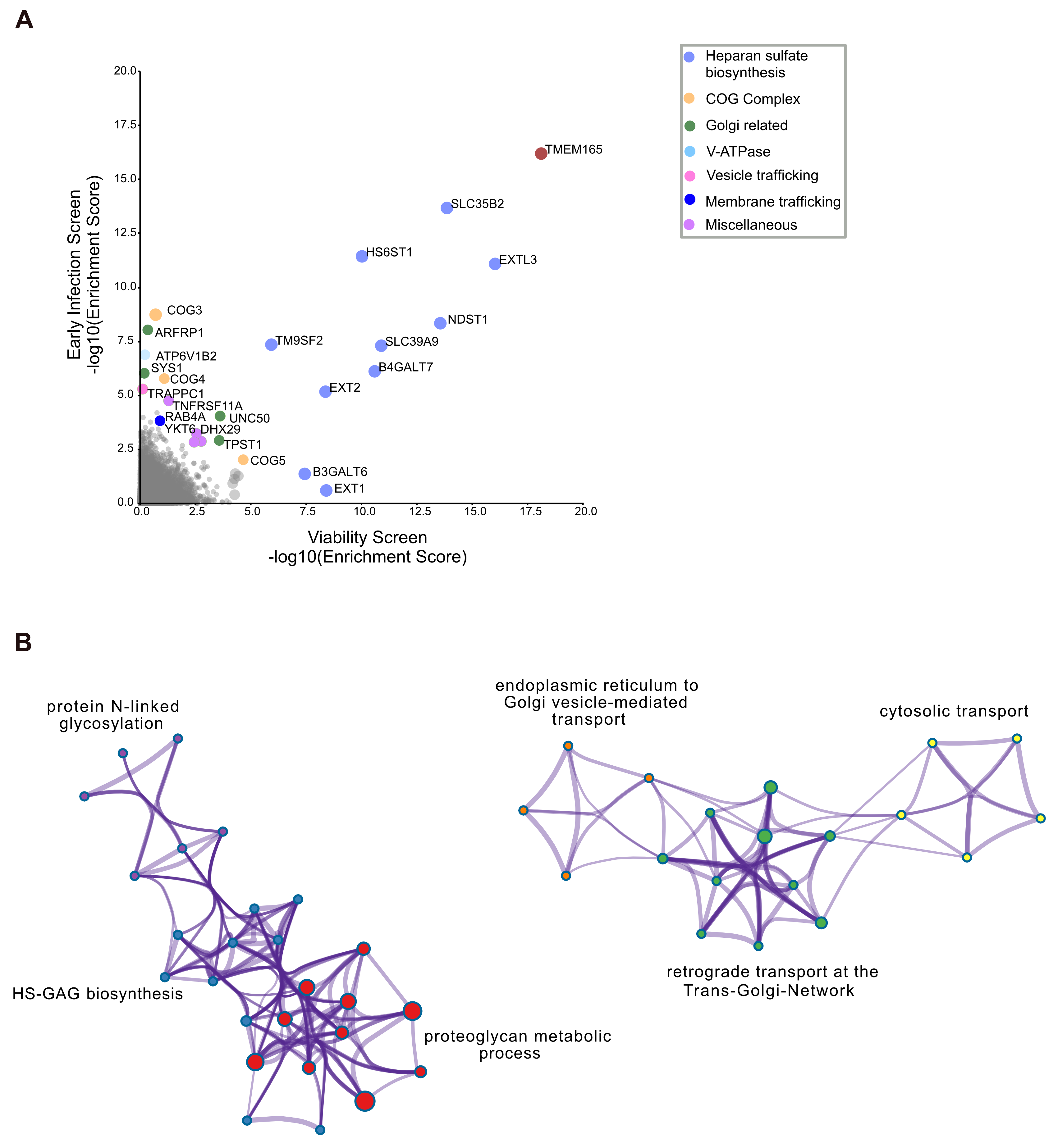


**Figure S4: RSV screen comparisons and pathway enrichment of results.**

**A.** We compared the results of our two genome-wide screens using the -log10 MAGeCK enrichment scores. Each gene is a dot and top genes are colored by function. **B.** Pathway and process enrichment clusters from our top genes were identified using Metascape (27). Each node in this network is an enriched term and nodes are colored by biological cluster description.


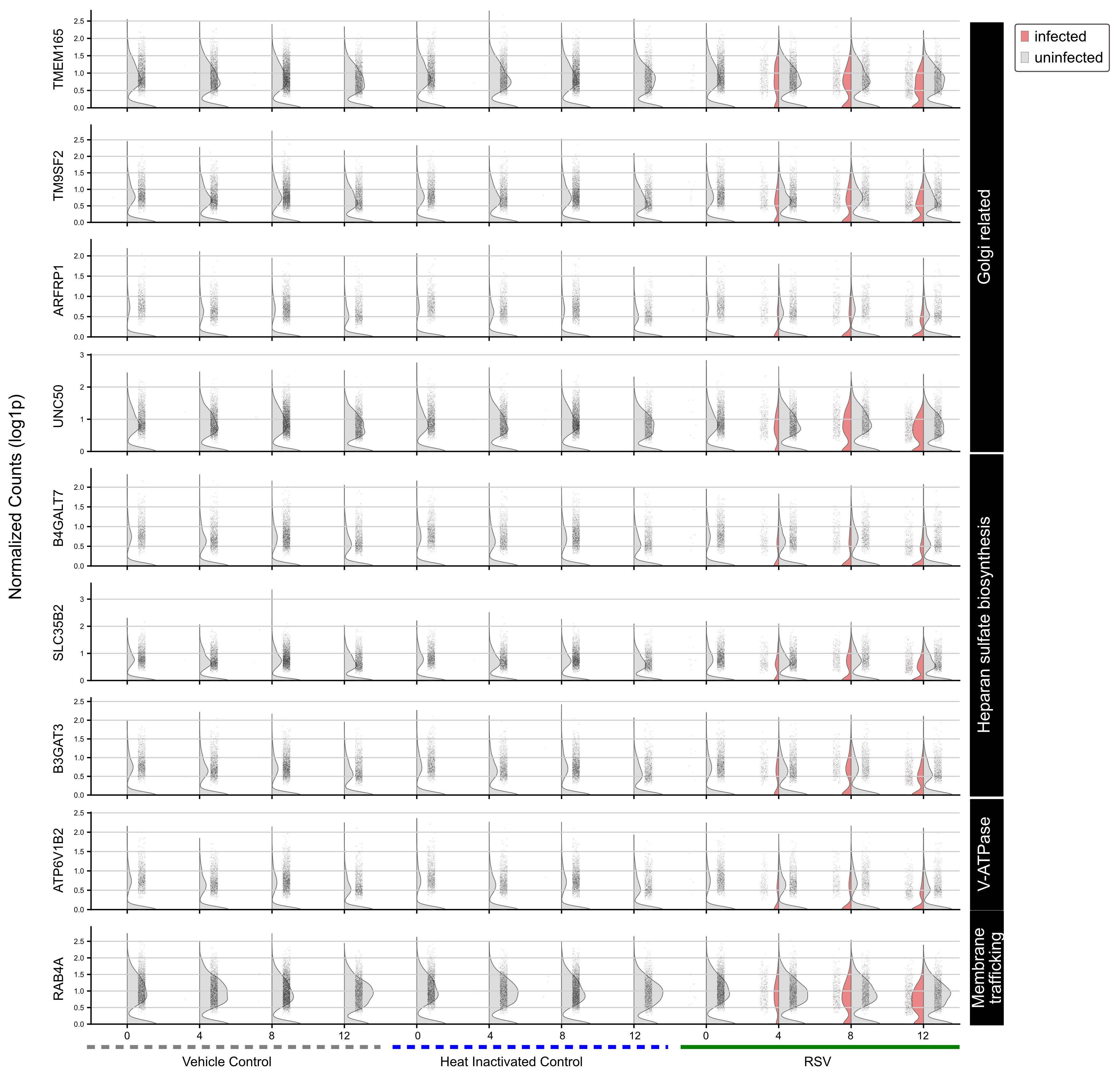


**Figure S5: Transcriptional expression of CRISPR screening hits.**

We evaluated the transcriptional expression of selected CRISPR screening hits in our single-cell time course of RSV infection. For each treatment condition and time point, the violin plot displays the normalized counts (log1p) for a given gene. Each violin is split by infection status (infected in pink and uninfected in gray) and every dot represents a single cell. Genes were selected based on the criteria that expression is at least 5 counts in a minimum of 10 cells in the dataset. For each time point, there was no significant difference (log2 fold change > 1, padj < 0.05) in expression for these genes when compared to the vehicle control.


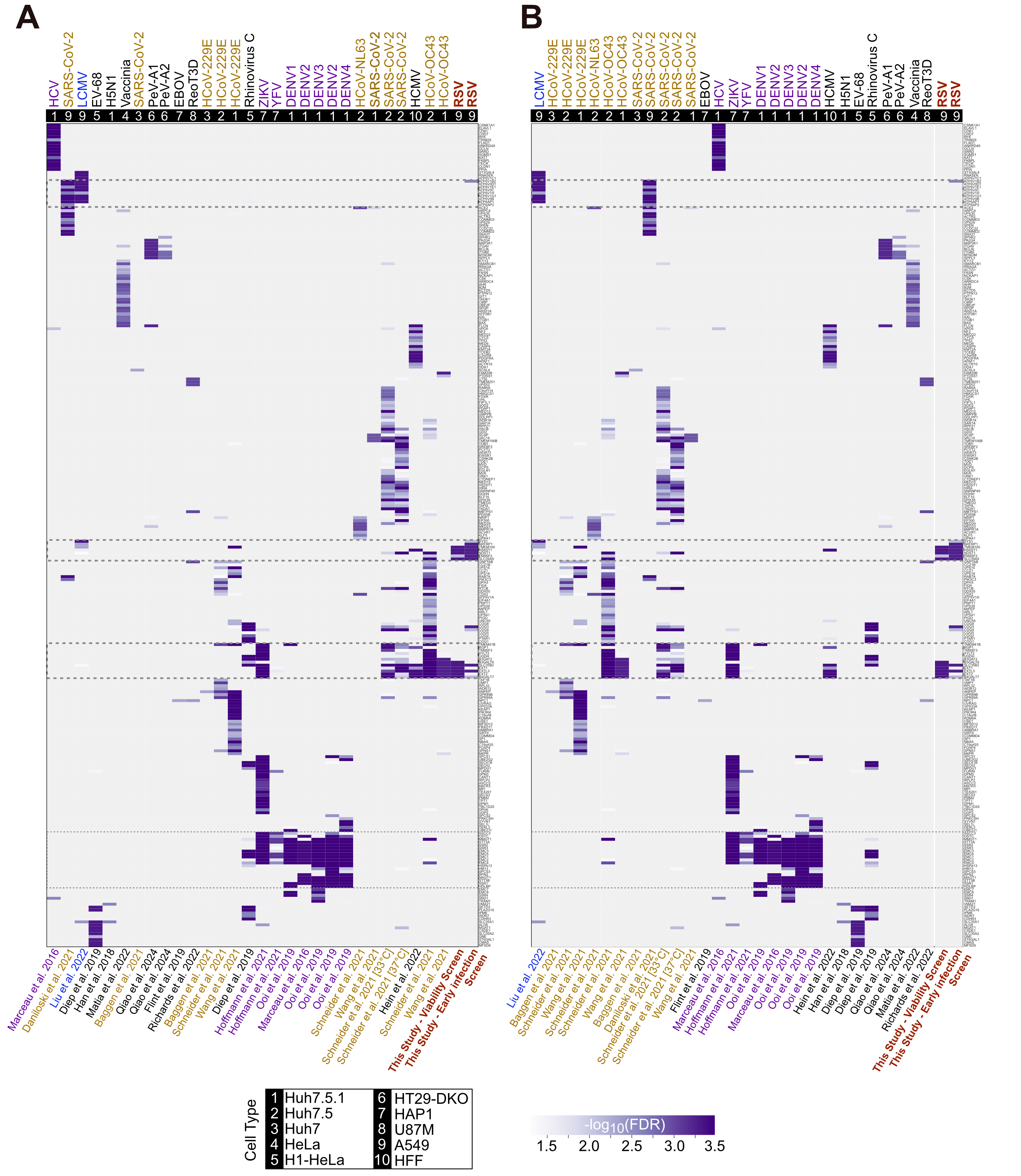


**Figure S6: Contextualization of RSV screening results.**

**A**. Expanded heatmap from Figure 4 detailed with all genes and associated study identifiers. Comparative screening results were replotted and grouped by virus family and virus (**B**).
