# Supplementary material for: Defining the host dependencies and the transcriptional landscape of RSV infection
Source: mBio. 2025 Aug 15;16(9):e01010-25. doi: 10.1128/mbio.01010-25 (PMC12421885; doi:10.1128/mbio.01010-25)
Supplement: Legends — for supplemental material. [file mbio.01010-25-s0003.docx]

**Table S1:** Results from differential expression analysis for each time point and condition.

**Table S2:** Genome-wide CRISPR screening results for RSV screens using MAGeCK enrichment analysis.

**Table S3:** Comparison of RSV screening results with 29 published CRISPR screens. These data are plotted in Figure 4.

**File S1:** Consensus genome for respiratory syncytial virus utilized in single-cell RNA sequencing experiments.
